# Exploring the Healing Potential of Aqueous Extract of *Baccaurea ramiflora* Leaves in Inflammation: A Cytokine and Prostaglandin Modulator

**DOI:** 10.1002/fsn3.70356

**Published:** 2025-05-23

**Authors:** Muhammad Nasir Hayat Malik, Md. Mahedi Hassan Tusher, Begum Rokeya, Sidra Javaid, Saud O. Alshammari, Amr S. Abouzied, Qamar A. Alshammari, Abdulkarim Alshammari, Maryam Zaib, Hafiz Muhammad Bilal, Muhammad Atif, Gideon F. B. Solre

**Affiliations:** ^1^ Faculty of Pharmacy The University of Lahore Lahore Pakistan; ^2^ Department of Pharmacology, Faculty of Basic Sciences Bangladesh University of Health Sciences (BUHS) Dhaka Bangladesh; ^3^ Rashid Latif College of Pharmacy Rashid Latif Khan University Lahore Pakistan; ^4^ Department of Pharmacognosy and Alternative Medicine, College of Pharmacy Northern Border University Rafha Saudi Arabia; ^5^ Department of Pharmaceutical Chemistry College of Pharmacy, University of Hail Hail Saudi Arabia; ^6^ Department of Pharmacology and Toxicology, College of Pharmacy Northern Border University Rafha Saudi Arabia; ^7^ Department of Pharmacy Practice, College of Pharmacy Northern Border University Rafha Saudi Arabia; ^8^ Department of Chemistry, Thomas J. R. Faulkner College of Science and Technology University of Liberia Monrovia Montserrado County Liberia

**Keywords:** antioxidant, BrLAE, CFA, cytokines, inflammation, prostaglandins

## Abstract

*Baccaurea ramiflora* is a common edible nutritious plant that has been associated with a variety of medicinal attributes. This study investigated the anti‐inflammatory effects of the aqueous extract of *B. ramiflora* leaves (BrLAE) in a complete Freund's adjuvant (CFA) induced inflammatory model. BrLAE was prepared by the cold maceration process and screened for phytochemical and antioxidant analyses. Inflammation was induced by a single intra‐planter (i.pl.) injection of CFA (100 μL) in the hind paws of male Wistar rats. After 7 days of immunization, paw edema was measured weekly (Day 7–Day 35) by digital plethysmometer and vernier caliper. Animals were daily treated with either methotrexate (MTX) or BrLAE (300, 600, and 1000 mg/kg) from Day 7 to Day 35. At the end of the experimental period, blood and liver samples were collected for the analysis of hematological, biochemical parameters and gene expression, respectively. The findings revealed that BrLAE had significant antioxidant activity and a remarkably high amount of tannins, saponins, flavonoids, phenols along with a trace amount of alkaloids, steroids, and terpinoids. BrLAE significantly reduced paw edema levels and improved hematological (RBC, WBC, Hb, PLT, and ESR) and biochemical (ALT, AST, and CRP) parameters. Moreover, BrLAE prominently downregulated mRNA levels of pro‐inflammatory mediators, such as *TNF‐α*, *IL‐1β*, *NF‐κB*, *COX‐2*, *PTGDS*, *mPGES1*, and *MMP1*. Molecular docking of the active constituent (Rosmarinic acid) with TNF‐α, NF‐κB, COX‐2, PTGDS, and mPGES1 also supported the experimental findings. These findings highlight that BrLAE possesses robust antioxidant and anti‐inflammatory properties which may be attributed to its inhibitory effects on prostaglandins and cytokines.

## Graphical abstract

This study demonstrates that the aqueous extract of *Baccaurea ramiflora* leaves (BrLAE) possesses significant antioxidant and anti‐inflammatory properties. BrLAE treatment effectively reduced CFA‐induced paw edema, improved hematological and biochemical parameters, and downregulated pro‐inflammatory gene expression in Wistar rats. Molecular docking analysis further supported the inhibitory effects of its active constituent, rosmarinic acid, on key inflammatory mediators like TNF‐α, NF‐κB, and COX‐2.

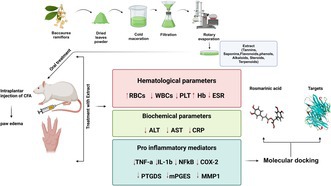

**Abbreviations**
ALT: alanine transaminase
AST: aspartate aminotransferase
BrLAE: aqueous extract of *Baccaurea ramiflora* leaves
CFA: complete Freund's adjuvant
COX‐2: cyclooxygenase‐2
CRP: C‐reactive protein
ESR: erythrocyte sedimentation rate
Hb: hemoglobin
IL‐1β: interleukin‐1 beta
MMP1: matrix metalloproteinase‐1
mPGES1: microsomal prostaglandin E synthase‐1
NF‐κB: nuclear factor kappa‐B
NO: nitric oxide
PLT: platelets
PTGDS: prostaglandin‐H2 D‐isomerase
RBC: red blood cells
ROS: reactive oxygen species
TNF‐α: tumor necrosis factor‐alpha
WBC: white blood cells

## 1 Introduction

Inflammation is a nonspecific immunological reaction of the body to foreign invaders. It is a crucial response of the host that facilitates the elimination of detrimental stimuli and repair of injured tissue for its survival and homeostasis (Ahmed 2011). The primary indicators of inflammation at the tissue level are edema, hyperthermia, erythema, pain, and functional impairment (Saleem et al. 2023). It is typically initiated within minutes in response to a stimulant, such as microbial infection, foreign invaders, or any irritant (external or internal). The selective expression of pro‐inflammatory molecules is mediated by several critical regulators involved in the entire inflammatory response process. Immune cells, including macrophages, dendritic cells, mast cells, neutrophils, and lymphocytes, are crucial to inflammatory responses (Akira et al. 2006). In addition to these immune cells, various nonimmune cells, including fibroblasts, endothelial cells, and epithelial cells, also contribute to inflammatory processes (Medzhitov 2010).

Macrophages are integral to the mammalian immune system, providing prompt protection against foreign entities. An increase in pro‐inflammatory cytokines, including tumor necrosis factor (TNF‐α), interleukin (IL)‐6, IL‐1β, nuclear factor‐kappa B (NF‐κB), and metabolic enzymes such as cyclooxygenases (COX) and lipoxygenase, has been seen, with a reduction in anti‐inflammatory cytokines, notably IL‐4 and IL‐10 (Saleem et al. 2020). Extended activation of immune cells results in increased nitric oxide generation as well as production of reactive oxygen species (ROS), which exacerbates the inflammatory response. The excessive generation of nitric oxide (NO) is mostly attributed to inducible nitric oxide synthase (iNOS). Overproduction of iNOS and ROS may result in the pathophysiology of acute disorders, such as septic shock, and chronic inflammatory diseases, including rheumatoid arthritis, atherosclerosis, chronic hepatitis, pulmonary fibrosis, and cancer. Consequently, NO generation mediated by iNOS in macrophages reflects the level of inflammation and may serve as a metric for evaluating the effects of medications or substances on inflammatory processes. Thus, the pursuit of innovative medications or chemicals that proficiently suppress the formation of ROS and NO has emerged as a viable therapeutic strategy for addressing inflammatory illnesses (Rukshala et al. 2021).

Nonsteroidal anti‐inflammatory medications are extensively used to alleviate inflammation. Nonetheless, these medications may induce several undesirable effects, including stomach irritation that may advance to gastric ulcers. Moreover, extended use of both steroidal and nonsteroidal anti‐inflammatory medications is linked to the onset of peptic ulcers. As a result, the quest for novel anti‐inflammatory and analgesic medications with reduced side effects has attracted the interest of healthcare professionals, dietitians, scientists, and medicinal chemists globally (Karbab et al. 2020).

Traditional medicines are the predominant source of natural medicines and herbal products in addition to being an indispensable source for developing anti‐inflammatory drugs (Aqeel et al. 2019; Meng et al. 2024; Turan et al. 2023; Yousaf et al. 2022). Scientists are tirelessly searching for new leads from traditionally known medicinal plants. Furthermore, according to the WHO estimation, 80% of the people in the world utilize traditional medicine for their primary health care needs, and the majority of the plant herbs are generally believed and considered safe for use against various ailments (Balkrishna et al. 2024; Qadir and Raja 2021). Bangladesh is a rich source of medicinal plants because of its geographical location and propitious climate. About 5700 angiosperms are growing in this country and over 500 are assumed to have medicinal properties. These plants produce a large number of phytochemical compounds which are most related to the treatment of inflammation (Hasan and Sultana 2018). Phytochemicals derived from plants may protect against free radicals damage, oxidative damage, and act as a supplementation of natural antioxidants in the human body (Boots et al. 2008). Studies report that plant‐derived flavonoids, alkaloids, and terpenoids are a rich source of antioxidant, anti‐inflammatory, and immunomodulatory activities (Abbas and Alkheraije 2023; Hussain et al. 2023).

*Baccaurea ramiflora* L., a member of the Phyllanthocin family (Local name: Lotkon), is a slow‐growing evergreen tree, often cultivated in gardens due to its edible fruit. The substantial levels of vitamin C, protein, and iron in *B. ramiflora* fruit contribute to its popularity. As ancient times, different components of *B. ramiflora* have been used to treat several ailments. The fruit has antiviral and antioxidant effects, whereas the leaves and stems may exhibit cytotoxic qualities (Arulselvan, Fard, et al. 2016). Moreover, phytochemical screening has revealed that *B. ramiflora* fruit is abundant in bioactive compounds, including alkaloids, tannins, flavonoids, terpenoids, proteins, carbohydrates, phenols, saponins, and fixed oils, which are advocated for various pharmacological activities, such as expectorant, immunomodulatory, vasoprotective, antioxidant, hypocholesterolemic, hypoglycemic, antifungal, and antiparasitic effects (Uddin et al. 2018). Based on the diverse pharmacological potential of *B. ramiflora* , this study was designed to mechanistically evaluate the anti‐inflammatory effects of the aqueous extract of *B. ramiflora* leaves (BrLAE).

## 2 Materials and Methods

### 2.1 Reagents and Chemicals

Complete Freund's adjuvant (CFA) and Trizol were obtained from Sigma‐Aldrich, Taufkirchen, Germany; Methotrexate from PharmaSol, Lahore, Pakistan; and the WizScript cDNA Synthesis Kit (High Capacity) and WizPure qPCR Master (SYBR) from Wizbio Solutions, New Mexico, USA. All other chemicals and reagents used in the study were of standard grade.

### 2.2 Collection of Plant Material

Leaves of *B. ramiflora* were gathered from Narsingdi, Bangladesh, and a voucher specimen was preserved in the National Herbarium of Bangladesh (Voucher specimen number: DACB 61280).

### 2.3 Preparation of BrLAE

The leaves of *B. ramiflora* were shade‐dried and ground into a fine powder using a grinding mill. The powder (1000 g) was extracted using distilled water over three cycles, and the extract was filtered using Whatman No. 1 filter paper. The filtrate was concentrated using a rotary vacuum evaporator at 40°C and dried using a freeze dryer. Approximately 165 g of a sticky brownish‐black dry extract was obtained from 1000 g of fine powder, and it was stored at 4°C for further experimental work. Percentage yield was calculated by using theformula:

$$\text{Percentage yield}=\frac{\text{Weight of extract}}{\text{Weight of sample}}\times 100$$

### 2.4 Phytochemical Analysis

Three grams of BrLAE was dissolved in 30 mL of distilled water. The heated sample solutions were subjected to filtration using Whatman No. 1 filter paper. The filtrate solution was examined for phytochemicals (alkaloids, tannins, phenols, terpenoids, steroids, saponins, and flavonoids) using the standard methods (Naseer et al. 2022).

### 2.5 DPPH Free Radical Scavenging Activity

The antioxidant activity was assessed by using the 2,2‐Diphenyl‐1‐picrylhydrazyl (DPPH) method. Different concentrations of the plant extract (50, 100, 200, 300, and 400 μg/mL) were prepared in 2 mL of methanol, and each concentration was mixed with 2 mL of DPPH (0.1 mM). Following a 30‐min incubation in darkness at ambient temperature, the absorbance was measured at the peak wavelength of 517 nm against standard ascorbic acid. The reduced absorbance of DPPH signifies an enhancement in DPPH radical scavenging activity. The scavenging potential was assessed by using the following formula:

$$I\%=\left[\left(A_{\text{blank}}-A_{\text{sample}}\right)/A_{\text{blank}}\right]\times 100$$

where, *I*% is the DPPH scavenging effect (%), *A*_blank_ is the absorbance of the control reaction (containing all reagents except the test compound), and *A*_sample_ is the absorbance of the test compound. The IC_50_ was the inhibition concentration that could scavenge 50% DPPH (Aqeel et al. 2019).

### 2.6 Complete Freund's Adjuvant (CFA) Induced Inflammation Model

Male albino Wistar rats (weighing 159–181 g) were procured from the University of Veterinary and Animal Sciences, Lahore, and housed in the animal care facility of the Faculty of Pharmacy, The University of Lahore, Lahore. They were kept for a period of acclimatization (7 days) at 22°C ± 3°C, a humidity of 40%–70%, and a 12‐h dark–light cycle, with access to a standard pellet diet (40% flour, 15% wheat bran, 8% maize bran, 4% rice bran, 10% fish meal, 3% beshon, 4% powdered milk, 0.5% salt, 1% oil, 1% vitamins, 0.5% molasses, and 8% oil cake) and water ad libitum. The experimental study was conducted according to ARRIVE guidelines, and approval was taken from the institutional research ethics committee of the Faculty of Pharmacy, The University of Lahore, Lahore (approval number: IREC‐FOP‐BAN‐01).

Inflammation was induced by administering a single dose of 100 μL CFA by intra‐plantar (i.pl.) injection. During the study period, body weight, paw edema, and joint diameter were measured at 7‐day intervals. Paw edema was assessed using a digital plethysmometer and digital vernier caliper (Patil and Patil 2017).

### 2.7 Experimental Design

Animals were divided into six groups with *n* = 6 in each group. For the evaluation of the anti‐inflammatory activity, all animals were treated for 35 days.

**Table:** 

Control groups (WC)	Animals received water (8.0 mL/kg)
Disease control group (DC)	Animals received water (8.0 mL/kg)
Methotrexate treated group	Animals received daily MTX (0.5 mg/kg)
BrLAE 300 mg/kg	Animals received daily BrLAE extract (300 mg/kg)
BrLAE 600 mg/kg	Animals received daily BrLAE extract (600 mg/kg)
BrLAE 1000 mg/kg	Animals received daily BrLAE extract (1000 mg/kg)

BrLAE doses were selected based on a previous study in which the alcoholic extract of *B. ramiflora* was used for its anti‐inflammatory potential (Saha et al. 2017).

### 2.8 Blood and Liver Sample Collection

At the end of the study period, animals were euthanized by i.pl. administration of pentobarbital sodium (200 mg/kg) (Laferriere and Pang 2020). A well‐trained laboratory technician euthanized the animals according to standard protocols. Blood samples were collected from the apex of the heart in gel tubes for biochemical analysis, and liver samples were extracted and preserved in Trizol reagent at −80°C freezer for mRNA analysis.

### 2.9 Hematological and Biochemical Test

Hematological parameters, including red blood cell count (RBC), white blood cell count (WBC), hemoglobin (Hb), platelet count (PLT), and erythrocyte sedimentation rate (ESR), were assessed using the Urit 3000 vet plus hematology analyzer. Biochemical parameters, specifically alanine transaminase (ALT) and aspartate aminotransferase (AST), were evaluated through calorimetric analysis employing standard ELISA kits (Cat # MBS264975, MBS269614). C‐reactive protein (CRP) was quantified using a latex agglutination assay. Quantitative determination was performed by multiple dilutions of blood serum and repeated agglutination reactions (Fernandes et al. 2012).

### 2.10 Real Time‐Quantitative Polymerase Chain Reaction (RT‐qPCR)

Liver samples were homogenized, and total RNA was extracted via standard Trizol protocol. RNA reverse transcription was conducted following the manufacturer's guidelines with the WizScript cDNA synthesis kit (Wizbio Solutions, New Mexico). The relative expressions of several genes were quantified using ΔΔCT technique and SYBR Green qPCR mix (Zokeyo, Wuhan, China). The RT‐PCR conditions consisted of an initial denaturation at 94°C for 2 min, followed by 40 cycles of denaturation at 94°C for 1 min, annealing at 60°C for 1 min, and extension at 72°C for 15 s. Hypoxanthine–guanine phosphoribosyltransferase (HPRT) mRNA served as a housekeeping control.

### 2.11 Molecular Docking Analyses

#### 2.11.1 Targets and Ligands Preparation

The three‐dimensional structures of the target proteins, including TNF‐α (PDB ID: 2az5), NF‐κB (PDB ID: 1IKN), COX2 (PDB ID: 5KIR), PTGDS (PDB ID: 3O19), and mPGES1 (PDB ID: 4yl3), were obtained from the Protein Data Bank (PDB). The receptor preparation involved several key steps: first, the PDB files were imported into MOE; then, unwanted chains, water molecules, and co‐crystallized ligands were removed to obtain clean receptor models. Following this, hydrogen atoms were added to ensure accurate docking simulations, and the protonation states of key residues were assigned according to the physiological pH relevant to the study (Saif et al. 2021).

The ligand structures, rosmarinic acid and MTX, were retrieved from the PubChem database, where their canonical SMILES were obtained. The ligands were later incorporated into MOE using the molecular builder option. The preparation steps included optimizing the ligands to obtain stable three‐dimensional conformations, followed by the assignment of partial charges using the AMBER force field to ensure accurate interaction modeling during the docking process (Mohamed et al. 2022).

#### 2.11.2 Docking Setup and Execution

Docking was configured using several key parameters. The MOE in‐built Site Finder tool was employed to identify the best binding pockets on the receptors, and a grid box was generated to encompass these regions. For the docking algorithm, the Triangle Matcher was selected for its efficiency in generating initial poses, followed by the Induced Fit protocol for further refinement of the docking results.

The docking simulation was initiated through the MOE interface, applying several key settings to ensure thorough exploration of ligand–receptor interactions. A total of 100 docking trials were conducted to ensure robust sampling of possible ligand orientations. The default scoring function in MOE was employed to evaluate the binding affinities of the generated poses, facilitating the identification of the most favorable ligand conformations within the receptor binding sites.

#### 2.11.3 Analysis of Docking Data

Post docking analyses were conducted to assess the results obtained from the simulations. The docked ligand conformations were visualized within the binding sites using MOE's visualization tools, as well as the Discovery Studio Visualizer for enhanced representation. Binding affinity evaluation involved comparing the binding scores to identify the most favorable poses. Additionally, an interaction analysis was performed to examine key interactions, including hydrogen bonds and hydrophobic contacts, providing insights into the binding mechanisms between the ligands and their respective receptors.

### 2.12 Statistics Analysis

Results were expressed as mean ± standard deviation (SD). Data of biological replicates were analyzed by one/two‐way analysis of variance (ANOVA) followed by post hoc test using Graph Pad Prism 8.02. *p* values of less than 0.05 were considered significant.

## 3 Results

### 3.1 Phytochemical Analysis

The preliminary phytochemical screening verified the presence of a high concentration of tannins, saponins, flavonoids, and phenolic compounds, along with trace amounts of alkaloids, steroids, and terpenoids in the extract (Table 1).

**TABLE 1:** Phytochemical screening of BrLAE.

Phytochemicals	BrLAE
Alkaloids	+
Tannins	+++
Saponins	+++
Flavonoids	+++
Phenols	+++
Steroids	+
Terpinoids	+

*Note:* +, Trace amount; +++, high amount.

### 3.2 BrLAE Reduced CFA Induced Oxidative Stress

The antioxidant capacity of BrLAE was determined using the DPPH free radical scavenging assay, a widely used method for evaluating the ability of a substance to neutralize free radicals. Free radicals are highly reactive molecules that can cause oxidative damage to cells and tissues, leading to various physiological disorders. The DPPH assay is based on the principle that the DPPH radical, which is deep purple in color, is reduced to a colorless or light yellow compound when it reacts with an antioxidant. The degree of discoloration is measured spectrophotometrically, and the results are expressed as IC_50_, which represents the concentration of the extract required to scavenge 50% of the free radicals. A lower IC_50_ value indicates stronger antioxidant potential, as it signifies that a smaller amount of the extract is needed to neutralize free radicals effectively. The results of the study showed that BrLAE exhibited significant free radical scavenging activity, with an IC_50_ value of 217.15 μg/mL. This suggests that the extract has a good capacity to counteract oxidative stress by donating electrons or hydrogen atoms to unstable free radicals, thereby preventing potential cellular damage. The antioxidant activity of BrLAE is likely attributed to the presence of bioactive phytochemicals, such as flavonoids, tannins, phenols, and saponins, which are known for their ability to act as natural antioxidants. These compounds can help stabilize free radicals and reduce oxidative stress by interrupting radical chain reactions. The observed IC_50_ value indicates that BrLAE has moderate to strong antioxidant properties, making it a potential natural source of antioxidants that could be beneficial for various applications, including health and medicinal research (Table 2).

**TABLE 2:** Antioxidant activity of BrLAE by DPPH method.

Ascorbic acid				BrLAE			
Conc. (μg/mL)	Cont. abs. (517 nm)	Abs.	% Inhibition	Conc. (μg/mL)	Cont. abs. (517 nm)	Abs.	% Inhibition
5	0.9790	0.9685	1.07	50	0.9790	0.79	19.35
10		0.8906	9.03	100		0.708	27.68
20		0.7093	27.55	200		0.505	48.41
40		0.3763	61.56	300		0.319	67.41
80		0.1071	89.06	400		0.189	80.69
IC_50_ (μg/mL)		41.4439		IC_50_ (μg/mL)		217.15	

### 3.3 Effects of BrLAE on Body Weight in CFA‐Induced Inflammatory Rats

**FIGURE 1:**
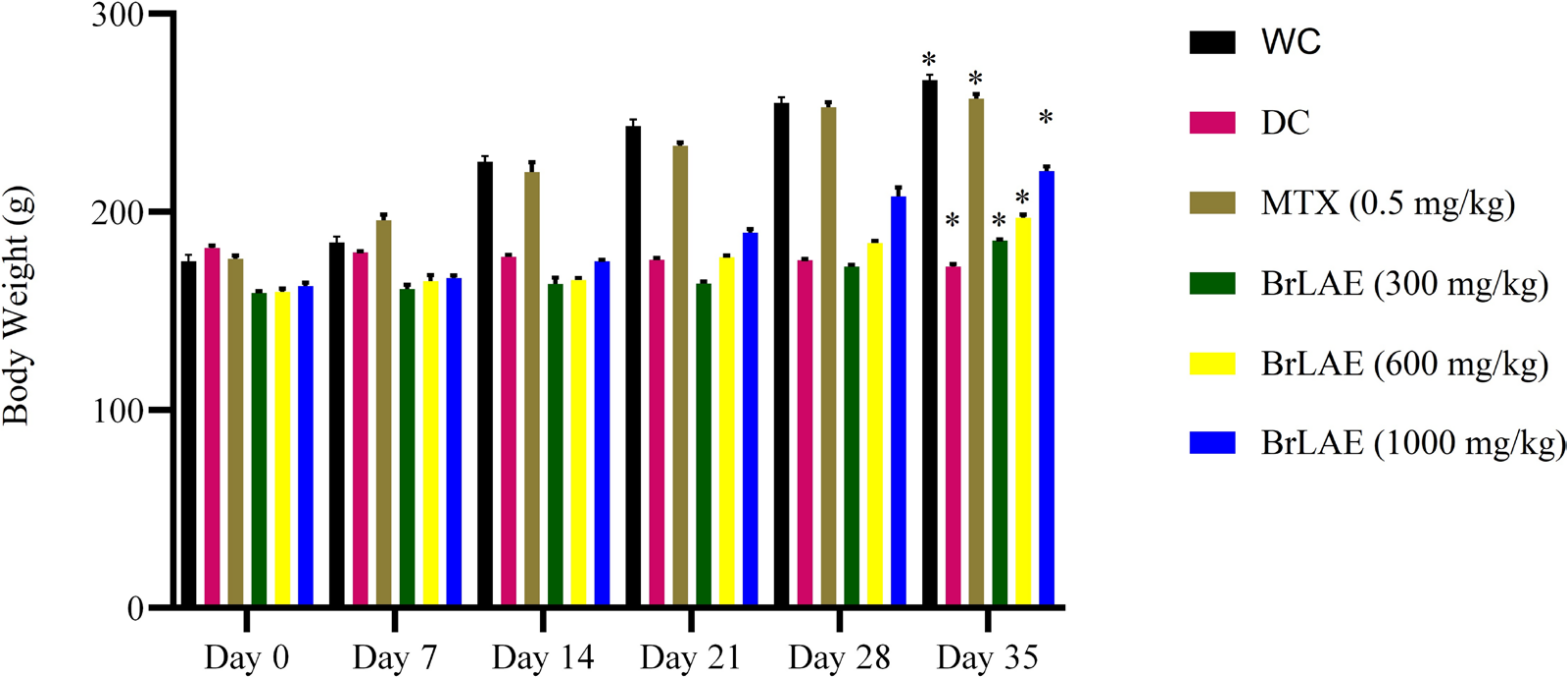
BrLAE restored CFA‐reduced body weight. Inflammation was induced by administering a single dose of (100 μL) CFA by intra‐planter (i.pl.) injection. MTX and BrLAE treated rats exhibited a significant increase in body weight on Day 35 as compared to DC group. Two way ANOVA followed by post hoc test, *p ≤ 0.05.

The body weight of each rat was measured weekly throughout the study to monitor the effects of inflammation and the administered treatments. Before the initiation of therapy, the baseline body weights of the rats varied slightly among different groups. The weight of the water control (WC) group was 175 ± 3.26 g, whereas the disease control (DC) group, which received CFA to induce inflammation, had an initial weight of 181 ± 1.25 g. The methotrexate (MTX) group, which received the standard anti‐inflammatory drug, had a body weight of 177 ± 2.00 g. The groups treated with BrLAE at different doses—300, 600, and 1000 mg/kg—had initial body weights of 159 ± 0.89, 159 ± 1.36, and 162 ± 1.95 g, respectively. These variations in initial weights indicate that while there were minor differences, the groups were relatively comparable at the beginning of the study. After 35 days of continuous therapy, significant changes in body weight were observed among the groups. The CFA‐treated DC group, which remained untreated, exhibited a 5% reduction in body weight, suggesting that chronic inflammation led to weight loss, likely due to metabolic stress, reduced food intake, or muscle wasting associated with inflammation. In contrast, the WC group (healthy control) showed a 51% increase in body weight, indicating normal growth and development in rats without inflammatory conditions. The MTX‐treated group exhibited a 44% increase in body weight, suggesting that despite its immunosuppressive effects, methotrexate helped maintain overall growth. Among the BrLAE‐treated groups, the 300 mg/kg dose resulted in a 16% increase, the 600 mg/kg dose led to a 23% increase, and the 1000 mg/kg dose produced a 35% increase in body weight. These findings suggest that BrLAE treatment, particularly at higher doses, contributed to a dose‐dependent improvement in body weight, possibly by reducing inflammation, improving appetite, or promoting metabolic balance (Figure 1).

### 3.4 BrLAE Significantly Reduced Paw Edema and Paw Diameter

CFA immunization led to severe paw inflammation in rats, except in the water control (WC) group, which remained unaffected throughout the study. Before any treatment was administered, paw edema levels varied among the different groups. The WC group, which did not receive CFA, had the lowest initial paw edema level at 2.49 ± 0.11 mL, whereas the disease control (DC) group, which was CFA‐treated but did not receive any therapeutic intervention, exhibited a significantly higher paw edema level of 5.25 ± 0.13 mL. The methotrexate (MTX)‐treated group had an initial paw edema level of 4.57 ± 0.35 mL, indicating moderate inflammation. Among the groups treated with BrLAE, the 300, 600, and 1000 mg/kg doses resulted in initial paw edema values of 5.33 ± 0.18, 4.68 ± 0.10, and 4.37 ± 0.10 mL, respectively. These baseline values suggest that all CFA‐treated groups developed significant inflammation before treatment began.

**FIGURE 2:**
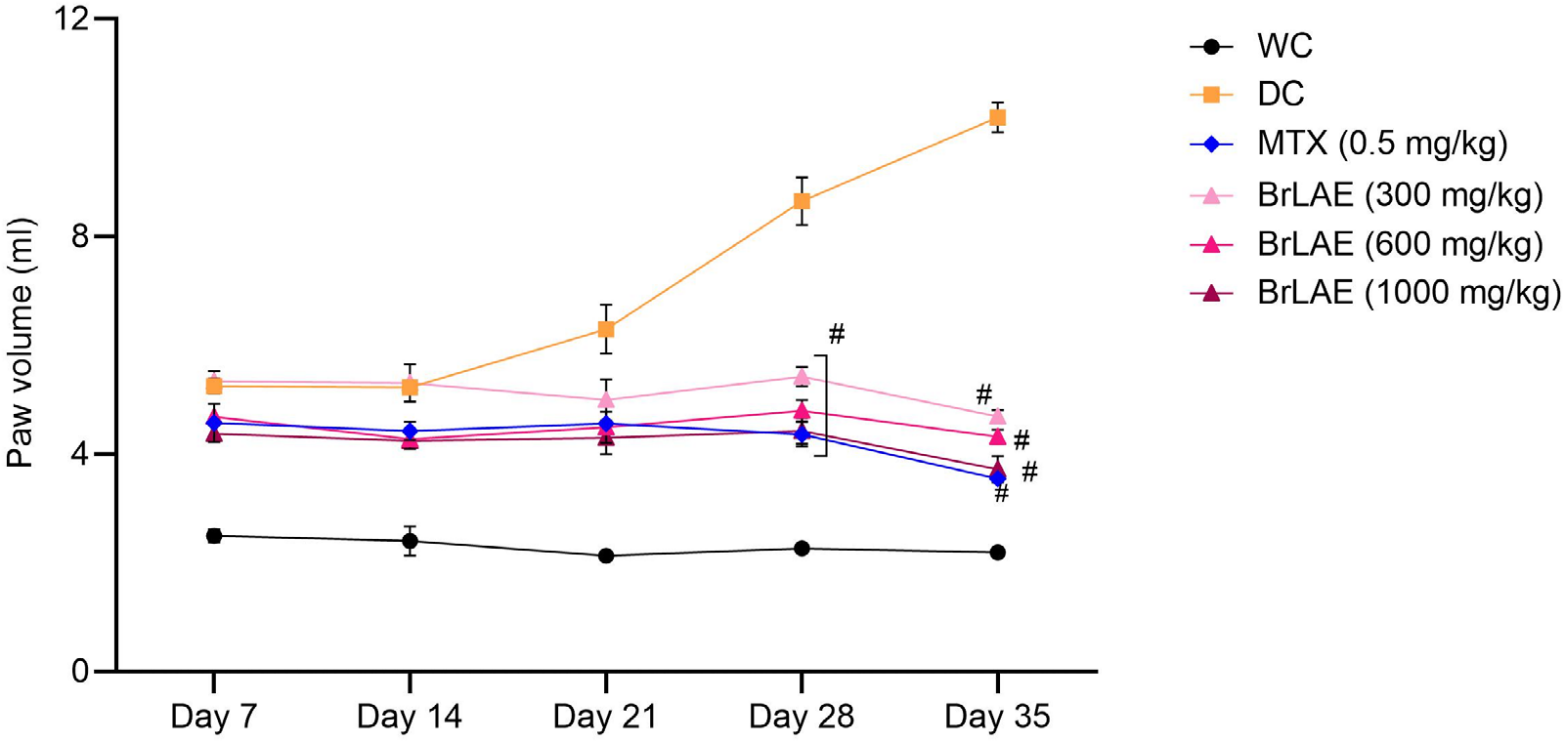
BrLAE reduced CFA‐induced paw edema. Inflammation was induced by administering a single dose of (100 μL) CFA by intra‐planter (i.pl.) injection. MTX and BrLAE treated rats exhibited a significant reduction in paw edema on Days 28 and 35. Two way ANOVA followed by post hoc test, ^#^ *p* ≤ 0.001.

Over the 35‐day treatment period, a significant reduction in paw edema was observed in all BrLAE‐treated groups. In the 300 mg/kg group, paw edema decreased from 6.33 ± 0.11 to 4.68 ± 0.11 mL, representing a 26% reduction. Similarly, the 600 mg/kg group showed a decrease from 5.95 ± 0.56 to 4.31 ± 0.13 mL, corresponding to a 27% reduction, whereas the 1000 mg/kg group exhibited the most notable reduction, from 5.48 ± 0.29 to 3.72 ± 0.24 mL, representing a 32% decrease. The MTX‐treated group, as expected, demonstrated a highly significant reduction in paw edema, from 7.36 ± 0.35 to 3.54 ± 0.04 mL, which corresponds to a 51% reduction (*p* < 0.001), confirming its effectiveness as a standard anti‐inflammatory drug. In contrast, the DC group, which did not receive any treatment, showed a steady progression of inflammation, with a 89% increase in paw swelling over the study period, along with noticeable hemorrhage and tissue damage. The WC group maintained normal conditions throughout the study without any signs of inflammation (Figure 2).

A similar trend was observed in the measurement of paw diameter. The BrLAE‐treated groups exhibited significant (*p* < 0.001) reductions in paw diameter over 35 days. In the 300 mg/kg group, paw diameter decreased from 7.72 ± 0.02 to 4.73 ± 0.16 mm, reflecting a 61% reduction. The 600 mg/kg group showed a similar effect, with paw diameter decreasing from 7.80 ± 0.31 to 4.90 ± 0.42 mm, representing a 62% reduction. The 1000 mg/kg group exhibited the highest reduction, from 6.59 ± 0.15 to 4.28 ± 0.20 mm, corresponding to a 64% decrease. In contrast, the DC group showed a worsening of inflammation, with paw diameter increasing by 80% compared to baseline values. These results indicate that BrLAE treatment significantly alleviated inflammation in a dose‐dependent manner, with higher doses producing greater anti‐inflammatory effects (Table 3).

**TABLE 3:** BrLAE reduced paw diameter of CFA‐treated rats.

Treatment groups	Paw diameter (mm)					
	0 day	7 days	14 days	21 days	28 days	35 days
WC	4.26 ± 0.06	4.21 ± 0.05	4.32 ± 0.04	4.89 ± 0.07	4.37 ± 0.04	4.59 ± 0.03
DC	5.26 ± 0.14	6.32 ± 0.01	6.31 ± 0.03	6.14 ± 0.04	9.30 ± 0.35	9.47 ± 0.32
MTX (0.5 mg/kg)	8.28 ± 0.04	5.57 ± 0.03	5.60 ± 0.05	5.82 ± 0.47	5.16 ± 0.08^#^	4.59 ± 0.29^#^
BrLAE (300 mg/kg)	7.72 ± 0.02	6.33 ± 0.04	6.36 ± 0.01	5.69 ± 0.31	4.79 ± 0.58^#^	4.73 ± 0.16^#^
BrLAE (600 mg/kg)	7.80 ± 0.31	5.35 ± 0.03	5.45 ± 0.03	5.26 ± 0.06	5.19 ± 0.11^#^	4.90 ± 0.42^#^
BrLAE (1000 mg/kg)	6.59 ± 0.15	5.28 ± 0.02	5.36 ± 0.03	5.51 ± 0.12	4.50 ± 0.38^#^	4.28 ± 0.20^#^

*Note:* Two way ANOVA followed by post hoc test.

^#^ *p* ≤ 0.001.

### 3.5 BrLAE Treated Rats Showed Improvement in Hematological Parameters

A marked elevation of WBC, PLT, and ESR was observed in CFA‐induced rats with a substantial decrease in RBC and Hb levels. In comparison to DC and MTX‐treated groups, BrLAE‐treated rats showed significant improvement in RBC and Hb levels, along with a significant decrease in WBC, PLT, and ESR levels. Moreover, BrLAE at 1000 mg/kg was more significant in improving RBC, WBC, PLT, ESR, and Hb levels compared to other treated groups, and these effects were even comparable to the standard MTX group (Table 4).

**TABLE 4:** BrLAE differentially modulated hematological parameters in CFA‐induced inflammatory rat model.

Treatment groups	RBC (×10^6^ μL)	Hb (g/dL)	WBC (×10^3^ μL)	PLT (×10^3^ μL)	ESR (mm/h)
WC	10.85 ± 0.21	17.65 ± 0.49	6.10 ± 0.28	837 ± 3.53	1.85 ± 0.07
DC	6.95 ± 0.50	11.16 ± 0.33	12.95 ± 0.63	982 ± 2.88	3.57 ± 0.20
MTX (0.5 mg/kg)	10.40 ± 0.29*	17.12 ± 0.17*	7.85 ± 0.81*	916 ± 2.82*	2.42 ± 0.22*
BrLAE (300 mg/kg)	9.26 ± 0.38*	13.37 ± 0.83*	8.50 ± 1.06*	953 ± 1.91*	2.91 ± 0.12*
BrLAE (600 mg/kg)	9.37 ± 0.35*	16.55 ± 0.47*	7.60 ± 0.47*	855 ± 1.91*	2.15 ± 0.12*
BrLAE (1000 mg/kg)	10.62 ± 0.34*	18.33 ± 0.10*	5.99 ± 0.21*	833 ± 2.50*	2.16 ± 0.11*

*Note:* One way ANOVA followed by post hoc test.

*p ≤ 0.05.

### 3.6 BrLAE Downregulated the Levels of CRP, AST, and ALT

Inflammation is normally associated with an elevation of acute‐phase inflammatory C‐reactive protein (CRP) as well as liver function markers (Sproston and Ashworth 2018). A marked elevation in the levels of CRP, ALT, and AST was observed in all CFA‐induced inflammatory rats as compared to the WC group. Treatment with MTX and BrLAE significantly reduced all these parameters compared to the DC group, as shown in Table 5. BrLAE‐treated group showed a dose‐dependent reduction in all parameters, and the results of the 1000 mg/kg dose were comparable to the standard drug (Table 5).

**TABLE 5:** BrLAE reduced the levels of CFA‐induced CRP, AST, and ALP.

Treatment groups	CRP (mg/L)	AST (U/L)	ALT (U/L)
WC	461 ± 0.70	75.50 ± 4.94	36.00 ± 4.24
DC	631 ± 4.96	163.50 ± 5.00	83.60 ± 1.69
MTX (0.5 mg/kg)	463 ± 5.18*	84.00 ± 2.44*	42.25 ± 2.62*
BrLAE (300 mg/kg)	531 ± 3.31*	63.25 ± 5.18*	33.25 ± 1.25*
BrLAE (600 mg/kg)	461 ± 3.86*	95.50 ± 1.91*	34.50 ± 1.73*
BrLAE (1000 mg/kg)	443 ± 2.66*	82.80 ± 2.16*	34.60 ± 2.96*

*p ≤ 0.05.

### 3.7 BrLAE Attenuated the Transcript Levels of Inflammatory Mediators

To understand the mechanism underlying the anti‐inflammatory potential of BrLAE, the expression levels of key inflammation‐related genes were analyzed using reverse transcription polymerase chain reaction (RT‐PCR). The genes selected for this study included tumor necrosis factor‐alpha (TNF‐α), nuclear factor kappa‐B (NF‐κB), interleukin‐1 beta (IL‐1β), cyclooxygenase‐2 (COX‐2), matrix metalloproteinase‐1 (MMP1), prostaglandin‐H2 D‐isomerase (PTGDS), and microsomal prostaglandin E synthase‐1 (mPGES1). These genes play crucial roles in inflammatory pathways, regulating processes, such as cytokine production, immune response activation, prostaglandin synthesis, and tissue remodeling. The transcript levels of these genes were measured in different treatment groups to evaluate how BrLAE influences inflammatory gene expression at the molecular level.

**FIGURE 3:**
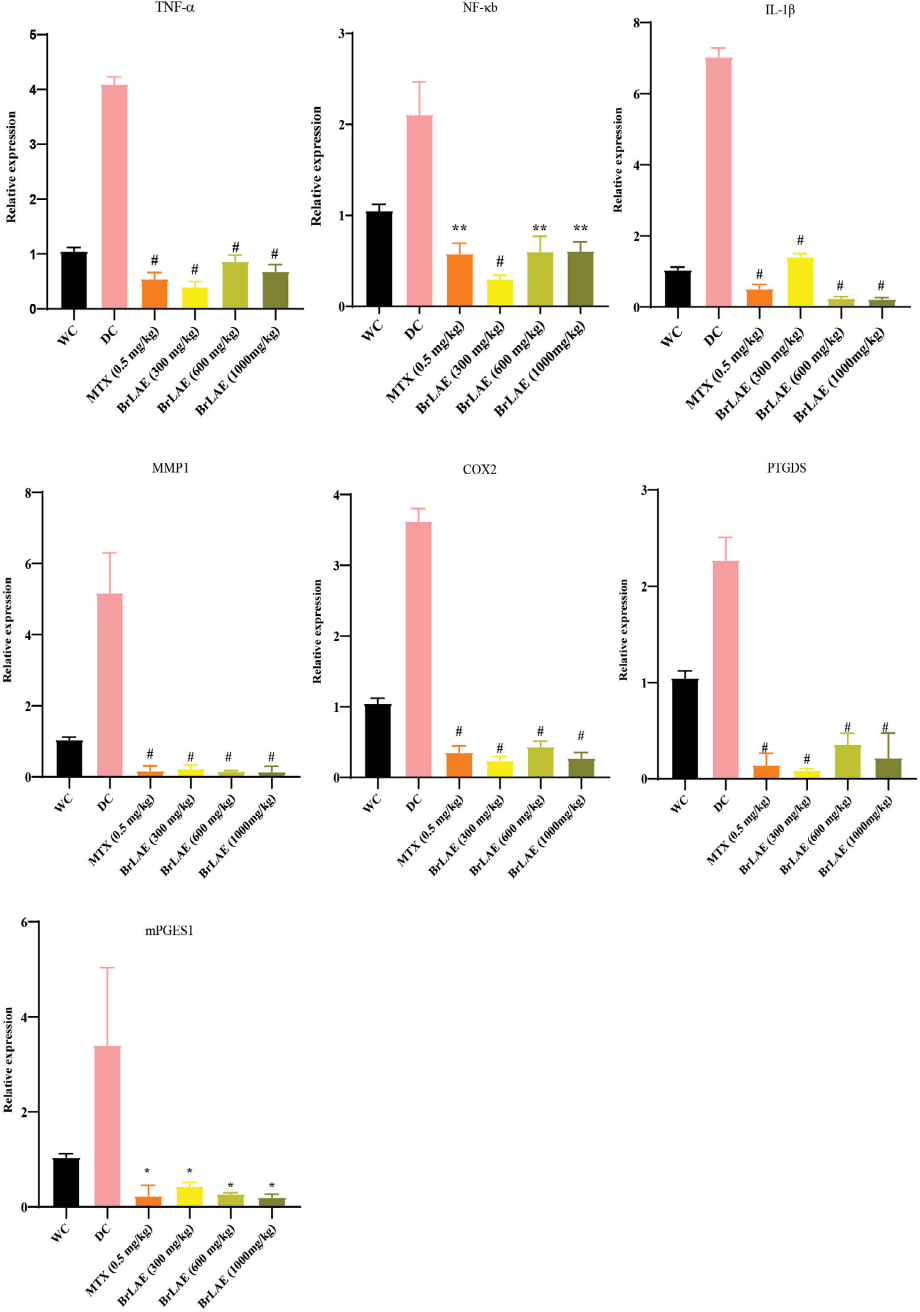
BrLAE reduced transcript levels of pro‐inflammatory mediators. Inflammation was induced by administering a single dose of (100 μL) CFA by intra‐planter (i.pl.) injection. MTX and BrLAE‐treated rats exhibited a significant reduction in mRNA levels of indicated genes. One way ANOVA followed by post hoc test, ^#^ *p* ≤ 0.001, **p ≤ 0.01, *p ≤ 0.05.

A significant increase in the genetic expression of these pro‐inflammatory mediators was observed in CFA‐induced inflammatory rats when compared to the normal (water control) group, indicating that CFA successfully triggered inflammation at the molecular level. The elevated expression of *TNF‐α*, *NF‐κB*, and *IL‐1β* suggests the activation of inflammatory signaling pathways, whereas increased *COX‐2*, *PTGDS*, and *mPGES1* expression indicates enhanced prostaglandin synthesis, which contributes to pain and swelling. Treatment with different doses of BrLAE resulted in a notable downregulation of these inflammatory markers, suggesting that the extract effectively suppressed the inflammatory response. The suppression was observed in a dose‐dependent manner, with higher doses showing stronger effects. These findings were comparable to the MTX‐treated group, which served as the standard anti‐inflammatory drug control, demonstrating that BrLAE exerts anti‐inflammatory effects similar to conventional therapeutic agents. The ability of BrLAE to modulate the expression of these genes suggests its potential to inhibit cytokine production, block prostaglandin synthesis, and prevent excessive tissue degradation, making it a promising natural candidate for managing inflammatory conditions (Figure 3).

### 3.8 Docking Affinities and Scores of Rosmarinic Acid With Target Proteins

**FIGURE 4:**
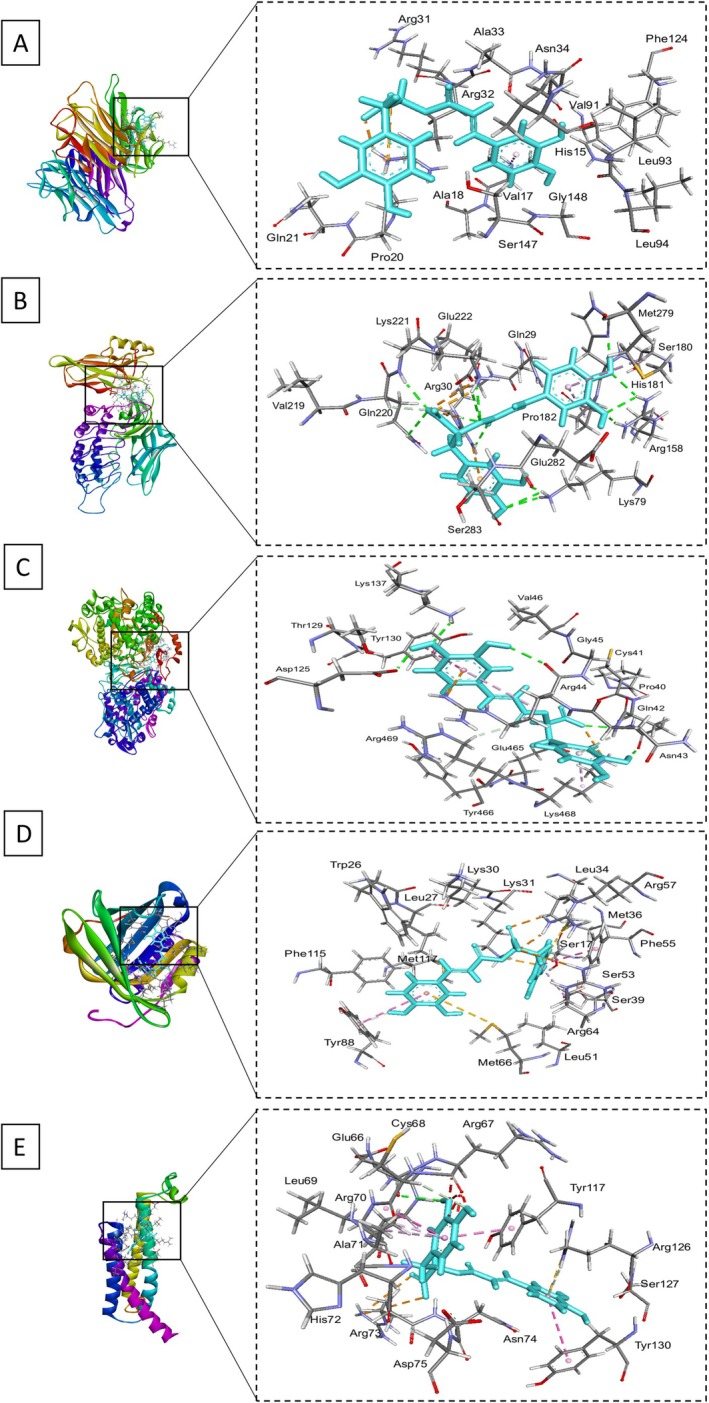
Docking conformations of rosmarinic acid (yellow) with target proteins. (A) TNF‐α, (B) NF‐κB, (C) COX2, (D) PTGDS, and (E) mPGES1. The figure highlights key binding interactions and critical residues. Each panel provides a detailed view of the ligand–protein complexes, emphasizing the distinct interaction patterns of both compounds within the respective binding sites.

**FIGURE 5:**
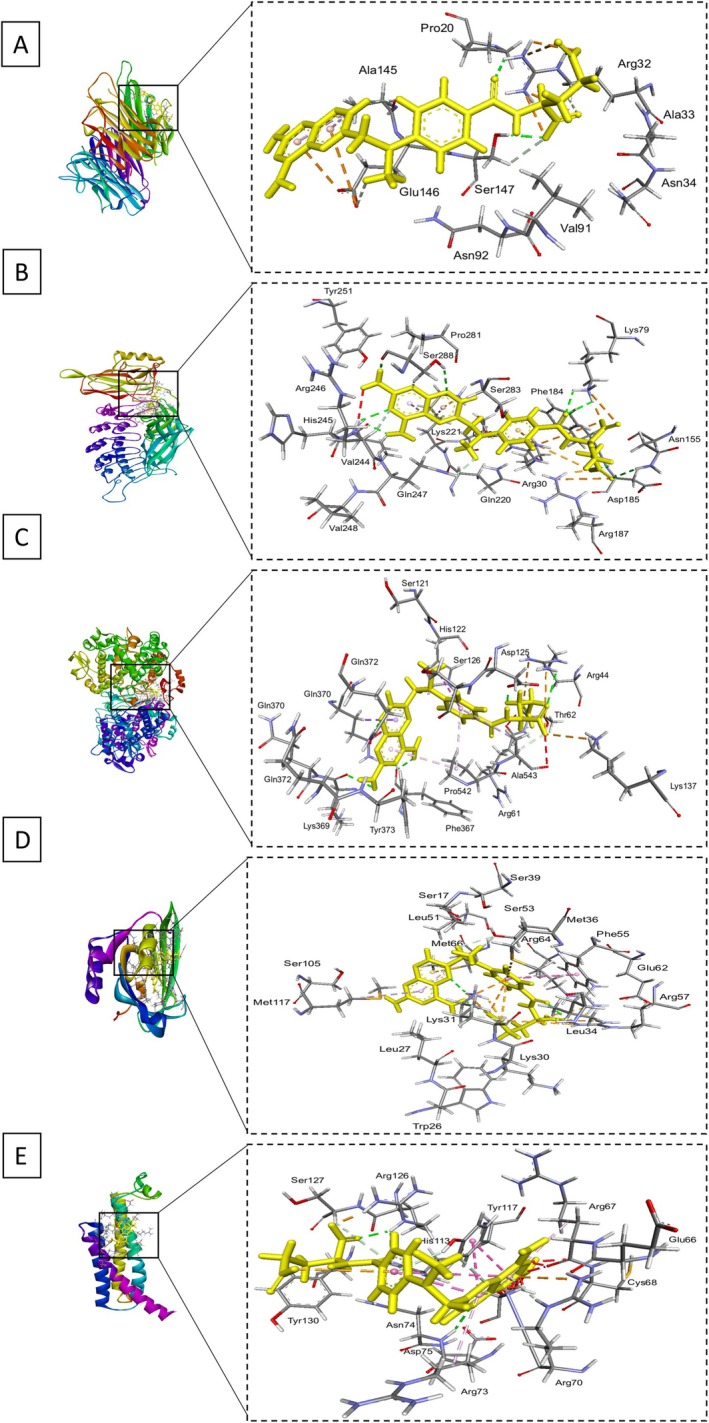
Docking conformations of MTX (yellow) with target proteins. (A) TNF‐α, (B) NF‐κB, (C) COX2, (D) PTGDS, and (E) mPGES1. The figure highlights key binding interactions and critical residues. Each panel provides a detailed view of the ligand–protein complexes, emphasizing the distinct interaction patterns of both compounds within the respective binding sites.

Studies have shown that the leaves of *B. ramiflora* contain bioactive compounds, such as rosmarinic acid, 6′‐O‐vanilloyltachioside, and 6′‐O‐vanilloylisotachioside, which exhibit significant anti‐inflammatory properties (Rohilla 2023). To further investigate their potential, we conducted molecular docking studies of rosmarinic acid with key molecular targets identified in in vivo experiments. This computational approach helps elucidate the binding interactions and mechanisms underlying its anti‐inflammatory effects. By analyzing these interactions, we aim to provide deeper insights into the therapeutic potential of *B. ramiflora* and its active constituents, contributing to the development of novel anti‐inflammatory agents derived from natural sources. The molecular docking studies for rosmarinic acid and MTX against five target proteins—TNF‐α (PDB ID: 2az5), NF‐κB (PDB ID: 1IKN), COX2 (PDB ID: 5KIR), PTGDS (PDB ID: 3O19), and mPGES1 (PDB ID: 4yl3)—revealed significant binding affinities and interaction profiles. For TNF‐α, rosmarinic acid scored −4.8787 with an RMSD of 2.0187 Å, forming hydrogen bonds with the NH2 group of ARG 32 at 2.85 Å and ionic interactions at 3.30 and 3.48 Å, resulting in interaction energies of −1.5 kcal/mol for hydrogen bonds and −2.7 kcal/mol for ionic interactions. MTX exhibited a stronger binding score of −5.3515 and an RMSD of 3.00 Å, establishing multiple hydrogen bonds with O33 and O29 at distances of 2.87 and 3.09 Å, respectively, and several ionic interactions with ARG 32, contributing to interaction energies of −1.9 kcal/mol for hydrogen bonds and −3.9 kcal/mol for ionic interactions. In NF‐κB, rosmarinic acid achieved a binding score of −7.6817 with an RMSD of 2.0362 Å, engaging in hydrogen bonding with ND1 of HIS 181 at 2.83 Å and multiple hydrogen bonds with LYS 221, indicating an overall interaction energy of −0.8 kcal/mol for hydrogen donors and −1.8 kcal/mol for hydrogen acceptors. MTX showed an even lower score of −8.2096 and an RMSD of 1.6617 Å, forming numerous hydrogen and ionic interactions, particularly with SER 288 and ARG 246, leading to an interaction energy of −3.5 kcal/mol for hydrogen donors. For COX2, rosmarinic acid scored −7.6814 with an RMSD of 1.6808 Å, establishing hydrogen bonds with ASP 125 and ARG 44, with interaction energies of −2.1 and −1.5 kcal/mol, respectively. MTX achieved a superior binding score of −8.7410 and an RMSD of 1.4739 Å, highlighting its strong binding capacity through various interactions, including hydrogen and ionic bonds with residues like LEU 366 and ARG 44, resulting in an overall interaction energy of −1.7 kcal/mol for hydrogen bonds. In PTGDS, rosmarinic acid scored −6.2412 with an RMSD of 2.0835 Å, interacting predominantly with MET 36 and ARG 57 through hydrogen bonds, contributing energies of −0.5 and −2.2 kcal/mol. MTX showed a competitive binding score of −7.6659 with an RMSD of 2.7065 Å, establishing multiple interactions with residues such as LYS 31 and ARG 64, resulting in interaction energies of −4.7 kcal/mol for hydrogen bonds. At last, in mPGES1, rosmarinic acid scored −4.2869 with an RMSD of 1.2627 Å, engaging in hydrogen bonding with ARG 70 and ARG 73, leading to energies of −0.5 and −2.7 kcal/mol. MTX achieved a binding score of −5.4034 with an RMSD of 1.7001 Å, forming significant hydrogen and ionic interactions with key residues like ASN 74 and ARG 126, reflecting its strong binding profile (Table S1; Figures 4 and 5).

The molecular docking results highlight the potential of rosmarinic acid, the active constituent of *B. ramiflora* , as a promising natural anti‐inflammatory agent. Its strong binding affinities with TNF‐α, NF‐κB, COX2, PTGDS, and mPGES1 suggest that it can effectively modulate key inflammatory pathways. The interactions with TNF‐α, particularly through hydrogen bonding with ARG 32, indicate its role in suppressing excessive inflammation, making *B. ramiflora* a valuable natural source for managing autoimmune diseases.

Rosmarinic acid's inhibition of NF‐κB, a critical transcription factor in immune responses, further emphasizes its potential in reducing chronic inflammation. Its strong interaction with COX2, which regulates prostaglandin synthesis, suggests it could serve as a natural alternative to NSAIDs with fewer side effects. The interactions with PTGDS and mPGES1 also indicate its ability to regulate prostaglandin metabolism, supporting its broader anti‐inflammatory properties.

These findings validate the traditional use of *B. ramiflora* in herbal medicine and highlight its potential in developing plant‐based therapeutics. With its multi‐target activity, rosmarinic acid offers a natural, effective, and safer alternative to synthetic anti‐inflammatory drugs, making *B. ramiflora* a promising candidate for further research in inflammation management.

## 4 Discussion

Plants have traditionally been regarded as a safe and effective option for treating inflammatory disorders due to their rich phytochemical composition (Choudhary et al. 2015). Natural products contain bioactive compounds that exhibit antioxidant, anti‐inflammatory, and immunomodulatory properties. Numerous studies have identified flavonoids, phenols, tannins, alkaloids, and saponins as key phytochemicals with potent anti‐inflammatory effects (Bai et al. 2021). These compounds modulate inflammation through various molecular pathways, including the suppression of pro‐inflammatory cytokines and chemokines, the upregulation of anti‐inflammatory mediators, and the inhibition of NF‐κB activation, nitric oxide (NO) production, and eicosanoid synthesis (Dinda et al. 2017).

BrLAE was found to be rich in tannins, saponins, flavonoids, and phenols, with smaller amounts of alkaloids, steroids, and terpenoids. These phytochemicals are known for their strong antioxidant and anti‐inflammatory activities (Li et al. 2021). Flavonoids and polyphenols exert anti‐inflammatory effects by inhibiting the production of pro‐inflammatory cytokines, NO, eicosanoids, and interfering with NF‐κB and activating protein‐1 (AP‐1) signaling pathways (Hughes et al. 2017). Alkaloids suppress neutrophil migration and reduce NLRP3 inflammasome activation, thereby lowering the expression of inflammatory markers. Terpenoids and flavonoids inhibit COX, leading to a decrease in inflammatory mediators (Paschke et al. 2013).

Oxidative stress is a key contributor to inflammatory diseases, including rheumatoid arthritis, cardiovascular diseases, and neurodegenerative disorders such as Alzheimer's disease (Gerber et al. 2002). The antioxidant assay in this study highlighted that BrLAE exhibited significant free radical scavenging activity, likely due to its high levels of flavonoids, phenols, and saponins. These phytochemicals help reduce oxidative stress by scavenging ROS, thereby mitigating inflammation (Goyal et al. 2013). Similar findings have been reported in studies investigating other plant extracts with rich polyphenolic content, demonstrating their protective effects against oxidative stress‐induced inflammatory damage (Rudrapal et al. 2022).

Weight loss is a critical indicator of disease severity, often associated with metabolic changes leading to muscle atrophy and decreased appetite (Munro and Capell 1997). Inflammatory conditions, including arthritis, trigger metabolic abnormalities that promote weight loss (Nasuti et al. 2019). In this study, the administration of BrLAE significantly increased body weight in treated groups, except for the DC group, further reinforcing its anti‐inflammatory potential. This aligns with findings from other studies where anti‐inflammatory treatments, such as polyphenol‐rich plant extracts, restored body weight in experimental models of inflammation (Rudrapal et al. 2022).

CFA‐induced paw inflammation is a widely used model for evaluating anti‐inflammatory pharmacotherapy, where an increase in paw diameter signifies inflammation intensity (Li et al. 2018). The results showed that CFA‐treated rats exhibited severe paw inflammation, which was significantly reduced following BrLAE treatment. This suggests that BrLAE mitigates inflammation by suppressing inflammatory mediators, such as histamine, prostaglandins, and pro‐inflammatory cytokines (Arulselvan, Fard, et al. 2016; Arulselvan, Tan, et al. 2016). Our findings corroborate the growing body of evidence supporting the therapeutic potential of natural compounds in rheumatoid arthritis management. Notably, Zhou et al. (2022) recently demonstrated that xanthorrhizol ameliorates oxidative stress and inflammation in the CFA‐induced arthritis rat model, paralleling our observations. Their study reported that xanthorrhizol suppressed key pro‐inflammatory cytokines and lipid peroxidation while enhancing antioxidant enzymes, mirroring the mechanisms identified in our current work (Zhou et al. 2022). Similarly, in line with our findings, Cellat et al. (2022) investigated the effects of safranal on an experimentally induced rheumatoid arthritis model in rats, demonstrating its anti‐inflammatory and antioxidant properties. Their study reported that safranal significantly reduced pro‐inflammatory cytokine levels (e.g., TNF‐α, IL‐6) and oxidative stress markers while improving histopathological joint damage. These results align with our observations regarding the modulation of inflammatory pathways by plant‐derived bioactive compounds (Cellat et al. 2022). Moreover, similar findings have been observed in studies investigating flavonoid‐rich extracts, which effectively reduce paw edema by modulating COX and prostaglandin synthesis (Saleem et al. 2023, 2020).

Inflammation significantly affects hematological parameters, leading to a reduction in RBC and Hb levels and an increase in WBC and PLT counts (Zhou et al. 2020). This study observed decreased RBC and Hb levels and elevated WBC, PLT, and CRP levels following CFA administration. Treatment with BrLAE and MTX significantly restored RBC and Hb levels while reducing WBC, PLT, and CRP levels compared to the DC group. This suggests that BrLAE exerts anti‐inflammatory effects by inhibiting pro‐inflammatory cytokines and preventing premature hemolysis (Reddy and Reddanna 2009). The ability of phytochemicals to restore hematological balance has also been demonstrated in other studies, reinforcing their role in mitigating inflammation‐induced hematological alterations (Paiva‐Martins et al. 2009).

Cytokines are key regulators of inflammation, with pro‐inflammatory cytokines, such as TNF‐α, IL‐1β, and IL‐6 playing a pivotal role in disease progression (Nisar et al. 2023). These cytokines activate the NF‐κB pathway, leading to the transcription of genes involved in inflammation, including COX‐2 and MMPs. COX‐2 is a crucial enzyme that catalyzes the conversion of arachidonic acid to prostaglandins, which are potent mediators of inflammation (Chen 2010). Elevated levels of prostaglandins contribute to pain, swelling, and tissue damage in inflammatory conditions. In this study, BrLAE significantly downregulated the mRNA expression of TNF‐α, NF‐κB, IL‐1β, COX‐2, MMP1, PTGDS, and mPGES1 compared to the DC group, indicating a broad anti‐inflammatory effect. The anti‐arthritic effects observed in our study are supported by growing evidence of natural compounds modulating key inflammatory pathways in arthritis models. Particularly relevant are the findings of Zeng et al. (2022), who demonstrated that specneuzhenide significantly ameliorated complete Freund's adjuvant‐induced arthritis in rats through dual modulation of the NF‐κB and HO‐1/Nrf‐2 pathways. Their work complements our results, showing similar downregulation of pro‐inflammatory markers (TNF‐α, IL‐6) and oxidative stress parameters, whereas upregulating antioxidant defenses (Zeng et al. 2022). Moreover, our findings are consistent with previous studies demonstrating that flavonoids, saponins, and terpenoids inhibit COX‐2 expression and prostaglandin synthesis, leading to reduced inflammation (Nissinen and Kahari 2014). For instance, studies on curcumin and resveratrol have shown that these polyphenols effectively suppress NF‐κB activation and reduce COX‐2 expression, thereby limiting prostaglandin‐induced inflammation (Siljehav et al. 2012). Furthermore, the ability of BrLAE to modulate cytokine levels aligns with studies investigating the anti‐inflammatory effects of other medicinal plants, such as *Withania somnifera* and *Boswellia serrata* , which have been reported to downregulate TNF‐α and IL‐1β expression (Pope and Choy 2021).

## 5 Conclusion

This study unveils the remarkable anti‐inflammatory and antioxidant potential of BrLAE, demonstrating its efficacy through both in vivo and in silico investigations. BrLAE significantly attenuated CFA‐induced inflammation by reducing paw edema, restoring hematological and biochemical balance, and downregulating key pro‐inflammatory mediators, including TNF‐α, NF‐κB, COX‐2, and IL‐1β. Molecular docking studies further reinforced these findings, revealing strong binding affinities of rosmarinic acid with critical inflammatory targets, underscoring its mechanistic role in cytokine and prostaglandin modulation.

The potent bioactivity of BrLAE, coupled with its natural origin and minimal toxicity, positions it as a promising candidate for novel anti‐inflammatory therapeutics. By offering a plant‐based alternative with multi‐targeted efficacy, *B. ramiflora* holds immense potential in bridging the gap between traditional medicine and modern pharmacology. Future research should focus on clinical evaluations and molecular elucidations to further establish its therapeutic value. This work contributes to the growing pursuit of nature‐derived interventions, reinforcing the significance of phytochemicals in combating inflammation and related disorders.

## Author Contributions

**Muhammad Nasir Hayat Malik:** conceptualization (equal), formal analysis (equal), investigation (equal), methodology (equal), project administration (equal), resources (equal), software (equal), supervision (equal), validation (equal), visualization (equal), writing – review and editing (equal). **Md. Mahedi Hassan Tusher:** data curation (equal), formal analysis (equal), investigation (equal), methodology (equal), software (equal), validation (equal), visualization (equal), writing – original draft (equal), writing – review and editing (equal). **Begum Rokeya:** formal analysis (equal), funding acquisition (equal), investigation (equal), methodology (equal), project administration (equal), resources (equal), software (equal), validation (equal), visualization (equal), writing – review and editing (equal). **Saud O. Alshammari:** data curation (equal), formal analysis (equal), investigation (equal), methodology (equal), software (equal), validation (equal), visualization (equal), writing – review and editing (equal). **Sidra Javaid:** data curation (equal), formal analysis (equal), investigation (equal), methodology (equal), resources (equal), validation (equal), visualization (equal), writing – review and editing (equal). **Amr S. Abouzied:** data curation (equal), formal analysis (equal), investigation (equal), methodology (equal), software (equal), validation (equal), visualization (equal), writing – review and editing (equal). **Qamar A. Alshammari:** data curation (equal), formal analysis (equal), investigation (equal), methodology (equal), software (equal), validation (equal), visualization (equal), writing – review and editing (equal). **Abdulkarim Alshammari:** data curation (equal), formal analysis (equal), investigation (equal), methodology (equal), software (equal), validation (equal), visualization (equal), writing – review and editing (equal). **Maryam Zaib:** data curation (equal), formal analysis (equal), investigation (equal), methodology (equal), resources (equal), software (equal), validation (equal), visualization (equal), writing – original draft (equal), writing – review and editing (equal). **Hafiz Muhammad Bilal:** data curation (equal), formal analysis (equal), investigation (equal), methodology (equal), software (equal), validation (equal), visualization (equal), writing – review and editing (equal). **Muhammad Atif:** data curation (equal), formal analysis (equal), investigation (equal), methodology (equal), resources (equal), software (equal), validation (equal), visualization (equal), writing – review and editing (equal). **Gideon F. B. Solre:** conceptualization (equal), data curation (equal), formal analysis (equal), investigation (equal), methodology (equal), project administration (equal), resources (equal), software (equal), validation (equal), visualization (equal), writing – review and editing (equal).

## Conflicts of Interest

The authors declare no conflicts of interest.

## Supporting information


**Table S1.** Molecular docking of rosmarinic acid and MTX with different targets.

## Data Availability

The datasets used and/or analyzed during the current study are available from the corresponding author on reasonable request.
