# Supplementary material for: Exploring the Healing Potential of Aqueous Extract of Baccaurea ramiflora Leaves in Inflammation: A Cytokine and Prostaglandin Modulator
Source: Food Sci Nutr. 2025 May 23;13(6):e70356. doi: 10.1002/fsn3.70356 (PMC12102493; doi:10.1002/fsn3.70356)
Supplement: Supplementary file 1 — Table S1. Molecular docking of rosmarinic acid and MTX with different targets. [file FSN3-13-e70356-s001.docx]

**Supplementary results**

**Table S1: Molecular docking of rosmarinic acid and MTX with different targets.**

| **Compounds** | **S score (kcal/mol)** | **RMSD**  **(A^o^ )** | **Atom of compounds** | **Atom of receptors** | **Residue of receptors** | **Type of interaction** | **Distance** | ***E* (kcal/mol)** |
| --- | --- | --- | --- | --- | --- | --- | --- | --- |
| **TNF-α (PDB ID: 2az5)** | | | | | | | | |
| Rosmarinic acid | -4.8787 | 2.0187 | O11  O10  O11  O11 | NH2  NE  NE  NH2 | ARG 32 (B)  ARG 32 (B)  ARG 32 (B)  ARG 32 (B) | H-acceptor  Ionic  Ionic  Ionic | 2.85  3.30  3.48  2.85 | -1.5  -2.7  -2.0  -5.6 |
| MTX | -5.3515 | 3.00 | \| O33 \| \| --- \| \| O29 \| \| O29 \| \| O32 \| \| O32 \| \| O33 \| \| 6-ring \| | \| OG \| \| --- \| \| NE \| \| NH2 \| \| NE \| \| NH1 \| \| NH1 \| \| N \| | \| SER \| 147 \| (B) \| \| --- \| --- \| --- \| \| ARG \| 32 \| (B) \| \| ARG \| 32 \| (B) \| \| ARG \| 32 \| (B) \| \| ARG \| 32 \| (B) \| \| ARG \| 32 \| (B) \| \| SER \| 147 \| (B) \| | \| H-acceptor \| \| --- \| \| Ionic \| \| Ionic \| \| Ionic \| \| Ionic \| \| Ionic \| \| pi-H \| | \| 2.87 \| \| --- \| \| 3.09 \| \| 3.35 \| \| 3.5 \| \| 3.79 \| \| 3.59 \| \| 4.32 \| | \| -1.9 \| \| --- \| \| -3.9 \| \| -2.5 \| \| -1.9 \| \| -1 \| \| -1.6 \| \| -1.2 \| |
| **NF-κB (PDB ID: 1IKN)** | | | | | | | | |
| Rosmarinic acid | -7.6817 | 2.0362 | \| O23 \| \| --- \| \| O10 \| \| O11 \| \| O14 \| \| O14 \| \| O10 \| \| O11 \| \| 6-ring \| | \| ND1 \| \| --- \| \| NZ \| \| N \| \| NH1 \| \| NH2 \| \| NZ \| \| NH1 \| \| NE2 \| | \| HIS \| 181 \| (A) \| \| --- \| --- \| --- \| \| LYS \| 221 \| (A) \| \| LYS \| 221 \| (A) \| \| ARG \| 30 \| (A) \| \| ARG \| 30 \| (A) \| \| LYS \| 221 \| (A) \| \| ARG \| 30 \| (A) \| \| GLN \| 29 \| (A) \| | \| H-donor \| \| --- \| \| H-acceptor \| \| H-acceptor \| \| H-acceptor \| \| H-acceptor \| \| Ionic \| \| Ionic \| \| pi-H \| | \| 2.83 \| \| --- \| \| 3.18 \| \| 3.17 \| \| 2.78 \| \| 2.94 \| \| 3.18 \| \| 3.87 \| \| 4.22 \| | \| -0.8 \| \| --- \| \| -1.8 \| \| -3.7 \| \| -1.7 \| \| -1.1 \| \| -3.4 \| \| -0.8 \| \| -1 \| |
| MTX | -8.2096 | 1.6617 | \| N14 \| \| --- \| \| N15 \| \| N6 \| \| N11 \| \| O29 \| \| O29 \| \| O30 \| \| O33 \| \| O29 \| \| O29 \| \| O30 \| \| O32 \| \| O33 \| | \| O \| \| --- \| \| O \| \| N \| \| N \| \| NE \| \| NH2 \| \| NE \| \| NZ \| \| NE \| \| NH2 \| \| NE \| \| NZ \| \| NZ \| | \| SER \| 288 \| (D) \| \| --- \| --- \| --- \| \| VAL \| 244 \| (A) \| \| SER \| 283 \| (D) \| \| ARG \| 246 \| (A) \| \| ARG \| 187 \| (A) \| \| ARG \| 187 \| (A) \| \| ARG \| 187 \| (A) \| \| LYS \| 79 \| (A) \| \| ARG \| 187 \| (A) \| \| ARG \| 187 \| (A) \| \| ARG \| 187 \| (A) \| \| LYS \| 79 \| (A) \| \| LYS \| 79 \| (A) \| | H-donor  H-donor  H-acceptor  H-acceptor  H-acceptor  H-acceptor  H-acceptor  H-acceptor  Ionic  Ionic  Ionic  Ionic  Ionic | \| 2.89 \| \| --- \| \| 2.94 \| \| 3.01 \| \| 3.15 \| \| 3.04 \| \| 2.87 \| \| 3.13 \| \| 2.87 \| \| 3.04 \| \| 2.87 \| \| 3.13 \| \| 3.58 \| \| 2.87 \| | \| -3.5 \| \| --- \| \| -1.3 \| \| -1.3 \| \| -1.3 \| \| -1.5 \| \| -3.9 \| \| -0.8 \| \| -6.4 \| \| -4.2 \| \| -5.4 \| \| -3.7 \| \| -1.6 \| \| -5.4 \| |
| **COX2 (PDB ID: 5KIR)** | | | | | | | | |
| Rosmarinic acid | -7.6814 | 1.6808 | O23  O24  O25  O11  6-ring | \| OD2 \| \| --- \| \| O \| \| OE1 \| \| NZ \| \| N \| | ASP 125 (A)  ARG 44 (A)  GLN 42 (A)  LYS 468 (A)  ARG 44 (A) | H-donor  H-donor  H-donor  Ionic  pi-H | 2.82  3.25  2.82  3.89  4.22 | -2.1  -1.5  -2  -0.7  -0.6 |
| MTX | -8.7410 | 1.4739 | N14  N15  N24  N6  O30  O32  O29  O32  O33  6-ring | O  O  OD1  NE2  CA  NZ  NZ  NZ  NH2  CB | LEU 366 (B)  PHE 367 (B)  ASP 125 (A)  GLN 372 (A)  THR 62 (A)  LYS 546 (B)  LYS 546 (B)  LYS 546 (B)  ARG 44 (A)  GLN 370 (A) | H-donor  H-donor  H-donor  H-acceptor  H-acceptor  H-acceptor  Ionic  Ionic  Ionic  pi-H | 3.16  3.17  3.01  3.06  3.3  2.79  3.03  2.79  2.97  3.95 | -1.7  -1.8  -3.1  -0.8  -1  -4.1  -4.3  -6.1  -4.7  -1.2 |
| **PTGDS (PDB ID: 3O19)** | | | | | | | | |
| Rosmarinic acid | -6.2412 | 2.0835 | C2  O24  O11  O10  O11 | SD  SD  NH2  NH2  NH2 | MET 36 (A)  MET 66 (A)  ARG 57 (A)  ARG 57 (A)  ARG 57 (A) | H-donor  H-donor  H-acceptor  Ionic  Ionic | 3.77  3.37  3.02  2.94  3.02 | -0.5  -2.2  -2.7  -4.9  -4.3 |
| MTX | -7.6659 | 2.7065 | N9  O30  O32  O33  O33  O33  O29  O30  O32  O32  O33  O33  O33  O33 | NZ  NZ  NE  NH1  NH2  NH2  NZ  NZ  NE  NH2  NH1  NH2  NE  NH2 | LYS 31 (A)  LYS 31 (A)  ARG 64 (A)  ARG 57 (A)  ARG 57 (A)  ARG 64 (A)  LYS 31 (A)  LYS 31 (A)  ARG 64 (A)  ARG 64 (A)  ARG 57 (A)  ARG 57 (A)  ARG 64 (A)  ARG 64 (A) | H-acceptor  H-acceptor  H-acceptor  H-acceptor  H-acceptor  H-acceptor  Ionic  Ionic  Ionic  Ionic  Ionic  Ionic  Ionic  Ionic | 3.43  3.15  2.92  3.08  2.94  3.02  3.13  3.15  2.92  3.91  3.08  2.94  3.28  3.02 | -1.2  -4.7  -3.8  -6  -8.1  -4.7  -3.6  -3.5  -5  -0.7  -4  -4.9  -2.8  -4.3 |
| **mPGES1(PDB ID: 4yl3)** | | | | | | | | |
| Rosmarinic acid | -4.2869 | 1.2627 | O14  O11  6-ring  6-ring | CD  NE  NE  6-ring | ARG 70 (A)  ARG 73 (A)  ARG 126 (A)  TYR 117 (A) | H-acceptor  Ionic  pi-cation  pi-pi | 3.2  3.31  4.02  3.88 | -0.5  -2.7  -0.6  0 |
| MTX | -5.4034 | 1.7001 | N6  O23  O32  O33  O33  O32  O32  O33  O33  6-ring | N  NE  NH2  NE  CA  NE  NH2  NE  NH2  6-ring | ASN 74 (A)  ARG 126 (A)  ARG 126 (A)  ARG 126 (A)  SER 127 (A)  ARG 126 (A)  ARG 126 (A)  ARG 126 (A)  ARG 126 (A)  TYR 117 (A) | H-acceptor  H-acceptor  H-acceptor  H-acceptor  H-acceptor  Ionic  Ionic  Ionic  Ionic  pi-pi | 3.32  3.08  2.9  2.96  3.38  3.76  2.9  2.96  3.58  3.79 | -2.4  -1  -3.8  -1.7  -0.5  -1  -5.2  -4.7  -1.6  0 |
